# Evaluation Method of the Band Saw Blade Wear State Based on Current Signals

**DOI:** 10.3390/ma19091853

**Published:** 2026-04-30

**Authors:** Dongliang Li, Bing Chen, Jiahao Fu, Zihao Liu, Junchang Liu, Wenchu Ou, Guoyue Liu

**Affiliations:** 1 Hunan Provincial Key Laboratory of High Efficiency and Precision Machining of Difficult-to-Cut Material, College of Mechanical Engineering, Hunan University of Science and Technology, Xiangtan 411201, China; lidongliang8165@163.com (D.L.); f1998jh@outlook.com (J.F.); sheepgrain@163.com (Z.L.); liujc332@163.com (J.L.); ougangwenchu318@163.com (W.O.); 2 Bichamp Cutting Technology (Hunan) Co., Ltd., Changsha 410200, China; liuguoyue@bichamp.com

**Keywords:** band sawing, current signal, wear state, sawing force model

## Abstract

The band saw blade is distinguished by its multi-point and flexible cutting capability when sawing materials. Its wear form is significantly more intricate than that of traditional cutting tools, such as the lathe tool and the milling cutter. Preliminary experimental observations suggest a close correlation between the wear of band saw blades and the motor current of the driving wheel. Therefore, this study evaluates the wear condition of band saw blades using current signals. A mathematical correlation model was established between the driving wheel motor current signals and the load on the band saw. A comprehensive experimental study was conducted on the band saw blade, encompassing the entire lifecycle of sawing operations. The average wear width of the tooth tip was utilized as an indicator of tooth wear, and an investigation was conducted into the correlation between the driving wheel motor current signals and the wear state. The findings indicated that the driving wheel motor current signals could be utilized to assess the blade wear state with high precision, which would facilitate proactive maintenance and replacement strategies to optimize band saw performance and service life.

## 1. Introduction

During the sawing process, the band saw blade is subjected to the combined action of multiple complex stresses, such as cyclic tension, impact, bending, and torsion, among others. Therefore, the main failure modes are fatigue fracture affecting the back spring steel, together with wear of the tooth material, as documented in references [1,2,3]. It is worth mentioning that the band saw blade as a one-time tool, and restoring its sawing capability through resharpening becomes a challenge once tooth wear occurs. In the course of sawing, excessive tooth wear may induce abnormal vibrations in the band saw, resulting in chipping, beveling, and other malfunctions. These failures have the potential not only to compromise the processing quality and efficiency but also endanger the band saw equipment and valuable materials. Furthermore, they could pose a threat to the safety of personnel, ultimately leading to significant economic and safety concerns, as stated in reference [4].

Currently, both domestic and international researchers mainly focus on monitoring tool wear through various signals, such as cutting force, torque, power, vibration, acoustic emission, speed, and temperature, among others. This approach constitutes indirect monitoring. Although the accuracy of indirect monitoring methods may slightly lag behind that of direct monitoring techniques, they can generally meet the requirements in online monitoring of the wear condition of band saw blade’s teeth, particularly in cases where the precision requirements are not overly stringent. Sarwar et al. [5,6,7] found that both the thrust and cutting forces exerted on the band saw teeth increase with wear, albeit at different rates. This finding lays a theoretical foundation for monitoring band saw blade wear using cutting force. Liu Guoyue et al. [8] combined cutting force, feed force, and tension force for mathematical analysis, thereby establishing a simplified force model for the band saw blade teeth during the sawing process. Thaler et al. [9] conducted an exhaustive analysis of cutting force in band saw machining through experiments. Their findings indicated that the cutting force signal encapsulates information pertaining to material inhomogeneity within the sawed workpiece and unveils crucial characteristics of blade geometry linked to inhomogeneous wear of the band saw tooth. Additionally, discontinuities in the welding of the band saw tooth and blade are evident in the force signal. In the context of band sawing processes, chatter serves as an indicator of significant reductions in surface quality and tool life. Thaler et al. [10] introduced an acoustic-based online chatter detection method, which achieved a chatter detection success rate of over 96% after double cross-validation. Chen et al. [11] also designed an online platform dedicated to band saw blade wear monitoring. This platform utilizes acoustic emission signals generated by abnormal chatter of the band saw blade. By performing wavelet analysis on the acoustic signals produced during the sawing process of the band saw machine, the current wear state of the band saw blade teeth can be determined more accurately. Furthermore, Magno et al. [12] presented a novel low-power, cost-effective wireless accelerometer system for detecting band saw blade wear. This method employs an ultra-low-power sensor with a radio frequency triggering function, which activates an alarm when abnormal vibration is detected in the guide arm of the band saw machine. This significantly reduced the activity of the main network and facilitated monitoring tasks with lower energy consumption. It can be observed that during the cutting process, the tool temperature increases with the rise in cutting speed and feed rate. As the tool wears, its surface temperature rises to varying degrees, with the increase being particularly pronounced under severe wear conditions [13].

Although power and torque signals are commonly used to monitor tool wear, torque sensors typically need to be mounted on the rotating drive shaft of a band saw to perform this function. This requires modifications to the machine’s structure and is extremely costly. Power signals are susceptible to voltage fluctuations and nonlinear power factors, resulting in insufficient sensitivity during light-load cutting. In contrast, there is a direct physical mapping relationship between motor current signals and electromagnetic torque. Data acquisition requires no modifications to the machine tool spindle; it is a non-intrusive detection method; it offers high sampling resolution; and it responds more quickly to dynamic cutting loads [14].

Given that the band sawing process is nonlinear and the data are time-varying, it is difficult to generate accurate models with traditional identification methods, as emphasized in the references [15]. However, advancements in computer technology have facilitated the application of neural networks, fuzzy logic, and genetic algorithms in prediction of wear and monitoring of online machining processes. Saglam et al. [16] established a strongly nonlinear correlation between cutting parameters and tool wear, demonstrating the viability of artificial neural networks in creating tool wear prediction models for tool condition monitoring. Chen et al. [17] proposed a methodology for the control of feed rate, with a view to balancing the relationship between cutting efficiency and the life of the band saw blade. A constant-power sawing feed rate model was developed, which accounts for wear effects. This model enables dynamic adjustment of the feed rate based on the wear condition of the saw blade. In comparison with traditional constant-feed-rate methodologies, this strategy enhanced efficiency by 44%. The solution proposed is an effective response to the problem of saw tooth wear. Li et al. [18] presented a deep learning-based control strategy tailored to the band sawing process, where a model was first employed to generate a health index reflecting the band saw blade’s operational status. Subsequently, a convolutional neural network (CNN) model was employed to map the relationship between the band saw blade cutting parameters and the band saw blade tooth wear. The model then searched for the optimal cutting parameters. This control model determines optimal band sawing parameters determined by the tooth wear condition, thereby extending the service life of the bimetal band saw blade. Jourdan et al. [19] presented a condition monitoring system for bimetallic band saws based on computer vision. This system combines deep learning with conventional computer vision techniques, aiming to achieve a more accurate evaluation of band saw tooth wear conditions. In another study, Li et al. [20] explored the use of an integrated deep learning model for band saw tooth wear monitoring relying on audio sensors. This method employs audio denoising techniques such as fast Fourier transform (FFT), bandpass filtering, and decorrelation component analysis (DCA) to extract wear data from the machining process. Following this, various algorithms are utilized to train the integrated Convolutional Neural Network Detection Model, converting audio signals into audio images.

The utilization of high-precision sensors, including those employed for acoustic emission, vibration, or acoustics, is constrained by the substantial expenses associated with their installation and maintenance. Furthermore, these sensors are susceptible to the sawing environment, and their signals are easily subject to noise interference, which hinders their widespread adoption in the sawing industry. Consequently, there is an imperative to develop innovative monitoring methodologies that will propel the sawing industry towards a future of enhanced intelligence [21]. The present study establishes a relationship between the sawing force and the drive motor current, converting the sawing force into an equivalent current signal. The selection of the current signal as a means to monitor saw tooth wear offers several advantages, including low cost, simple installation, and high accuracy. Consequently, this approach is highly promising for practical application. The present study focuses on the construction of an experimental platform for sawing that integrates tooth wear measurement with current signal acquisition. The objective of this study is to utilize experimental data to comprehensively examine the intrinsic relationship between the drive motor current of the sawing machine and tooth wear. Additionally, this study aims to investigate the correlation between current and wear at various stages of wear. Consequently, a methodology was formulated to accurately ascertain the wear of high-speed steel bimetal band saw blades using drive motor current. The establishment of this method will provide a more precise and scientific basis for the operation and maintenance of band saws.

## 2. Current Signal and the Band Saw Tooth Wear Model

### 2.1. Experimental Platform

**Figure 1:**
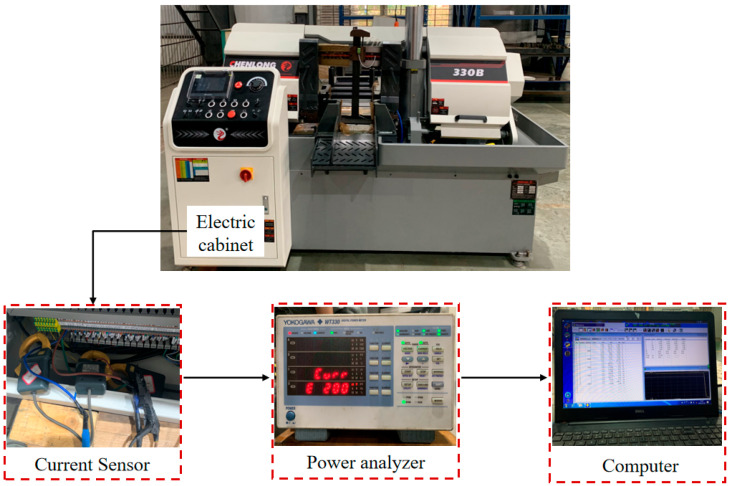
Band saw tooth wear experimental platform.

In this paper, a horizontal metal band saw machine equipped with a high-speed steel bimetal band saw blade is used to cut cylindrical workpieces, and a full lifecycle wear experiment is conducted on the band saw blade. The experimental platform used to study the wear of band saw blade teeth is shown in Figure 1. The steel workpiece to be cut is composed of 20# steel and is formed with a bar measuring 120 mm in diameter. By referring to the circuit diagram of the band saw machine, the three-phase power cable which corresponded to the drive wheel motor inside the electrical cabinet was identified. The cable was sequentially connected through the three ports of a Hall-type current sensor. This sensor used in this study underwent multi-point calibration using a precision current source prior to the experiment. The sensor’s linearity is better than 0.2%, and the measurement uncertainty is controlled within ±0.5% of full scale (FS). Additionally, a three-phase power analyzer was connected to the computer, with the current signals sampled at a frequency of 10 Hz. The wear of a band saw blade is a gradual physical process characterized by a long time scale. Furthermore, the drive system of a band saw has significant mechanical inertia, and the current envelope signals resulting from changes in cutting load are primarily concentrated in the low-frequency range. Experimental analysis indicates that a sampling rate of 10 Hz can meet the requirements for balancing data volume and computational efficiency in monitoring the service life of a band saw blade. The three-phase root mean square (RMS) current values were output to the computer through the acquisition card which was integrated into the instrument.

The present experiment was conducted on a Chenlong 330B intelligent horizontal band saw (manufactured by Shanghai Chenlong Sawing Machinery Co., Ltd., Shanghai, China), which possesses a drive motor with a rated power of 4.0 kW. The workpiece employed in this study was a 20# steel round bar with a diameter of 120 mm and a hardness of 170–250 HB. The cutting tool employed was a bimetal band saw blade (M42) with a blade width of 34 mm, a thickness of 1.1 mm, and a tooth pitch of 3/4 TPI (teeth per inch). The specifications, models, and the hardware devices parameters used in the experiment are listed in Table 1 below:

To assess the wear condition of the band saw teeth, the OST−FB201 optical microscope produced by OSTGUANGXUE is used to image the serrated flank with a magnification of 4 times. The most appropriate magnification was employed for the purpose of focusing on the back edge surface of the band saw teeth, particularly the area near the tooth tip. Subsequently, the microscope’s integrated measurement software was utilized to overlay a scale on the photographed image.

### 2.2. Analysis of Current Trend to Cutting Force

**Figure 2:**
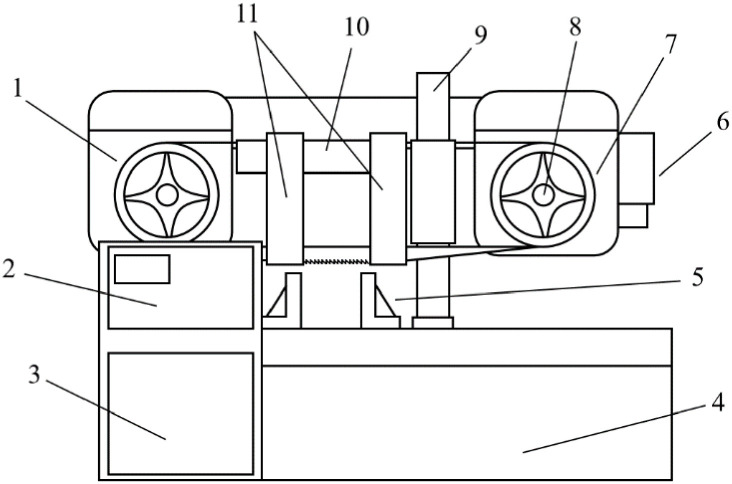
Structure of horizontal band saw machine. 1. Following Wheel. 2. Control Panel. 3. Electric Cabinet. 4. Chip Conveyor. 5. Front Clamp. 6. Drive Pulley. 7. Driving Wheel. 8. Driving Wheel Motor. 9. Column Tube. 10. Guideway. 11. Guide Arm.

The structure of the horizontal band saw machine, depicted in Figure 2, reveals that the band sawing process commences with the rotation of the saw wheel, during which the driving wheel motor remains in a no-load state. Upon contact between the band saw blade and the workpiece, the cutting force initially impacts the band saw tooth and subsequently transmits through the band saw blade, driving wheel, and transmission device to the driving wheel motor. This transmission results in a load variation, which manifests as a change in motor current. Primarily, the cutting force acts on the driving wheel in the form of torque. By synthesizing the aforementioned theories, the relationship between the driving wheel motor current and cutting force can be represented by the following Equation (1):

(1) $$K_{t}I_{rms}=T_{m}=J\frac{d\omega }{dt}+T_{f}+F_{x}R$$

where *K_t_* is the motor torque constant, *I_rms_* is the effective value of three-phase current of drive wheel motor, *T_m_* is the exerted on the driving wheel, *J* is the equivalent transmission inertia of the driving wheel drive system, *ω* is the angular frequency of the driving wheel, *T_f_* is the friction torque overcome by the transmission, and *F_x_* is the cutting force acting on the saw blade tangent to the driving wheel.

Since the driving wheel of the sawmill is powered by a three-phase AC asynchronous motor, assuming that the three-phase currents of the motor are *i_u_*, *i_v_*, and *i_w_*, then *I_rms_* is:

(2) $$I_{rms}=\sqrt{\left(i_{u}^{2}+i_{v}^{2}+i_{w}^{2}\right)/3}$$

Due to the unknown equivalent transmission inertia and friction torque in Equation (1), it is not possible to directly calculate the cutting force using the measured motor current of the driving wheel. During steady-state sawing, the main spindle speed fluctuation is less than ±1.5% because the band saw machine is equipped with a high-performance variable-frequency drive and a closed-loop PID control system. Furthermore, the high rotational inertia of the drive pulley effectively filters out speed fluctuations caused by transient load impacts. At the same time, the angular acceleration α = d*ω*/d*t* = 0, and the moment of inertia j is much smaller than the cutting resistance torque. It can be assumed that the angular velocity remains approximately constant, and the effects of inertia can be neglected. During empty cutting with the band saw blade, denoted as *F_x_*, we have [22]:

(3) $$K_{t}I_{rms0}=T_{f}$$

where *I_rms_*_0_ is the current at a certain line speed when the band saw blade is not cutting, called the no-load current.

Bringing Equation (3) into Equation (1) gives:

(4) $$\Delta I=F_{x}R/K_{t}$$

where ∆*I* = *I_rms_* − *I_rms_*_0_, represents the current increment relative to no-load operation when sawing the workpiece.

In Equation (14), let *K* = *R*/*K_t_*, then:

(5) $$\Delta I=KF_{x}$$

**Figure 3:**
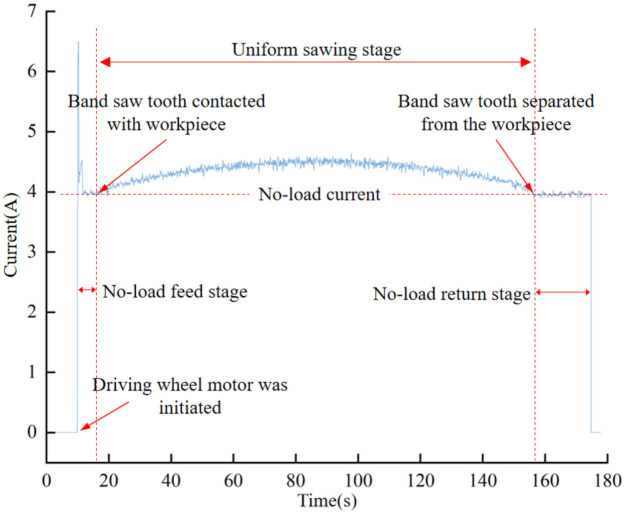
Variation of the root mean square (RMS) of motor current during the no-load, entry, and stable cutting phases when sawing a workpiece with a circular cross-section.

From the aforementioned reasoning, it becomes evident that there exists a linear relationship between the cutting force in the tangential direction of the driving wheel and the incremental current of the motor. Therefore, the magnitude of the incremental current of the driving wheel motor can be used as an indicator of the cutting force. As an increase in band saw tooth wear results in a greater cutting force, it is theoretically feasible to ascertain the state of the band saw tooth wear through the magnitude of the driving wheel motor current. When a band saw blade cuts a workpiece with a circular cross-section at a constant speed, the blade first approaches the workpiece while idling under no load; at this point, the drive motor maintains a roughly constant voltage. By recording the no-load current value before cutting begins, the current signal can be calibrated. In this experiment, the no-load current remained at approximately 4 A. as illustrated in Figure 3, which depicts current signal characteristics during different operational stages. Upon contact with the workpiece, the current initially increases and then decreases, with a trend that mirrors the width of the circular cross-section being sawed. Given that, under the same feed speed, a wider band saw blade experiences a greater cutting force, and the relationship between current and cutting force, as described by Equation (5), aligns with the aforementioned theoretical analysis, thus providing evidence of its reasonableness.

The utilization of the driving wheel motor current as an input signal not only offers technical feasibility but also numerous practical benefits. These advantages primarily relate to reduced monitoring expenses, simplified data handling, and enhanced data processing capabilities. Consequently, in this research, the current is employed as an indirect indicator of band saw tooth wear for subsequent data acquisition and analysis.

### 2.3. Analysis of Band Saw Tooth Wear Process and Wear Amount

**Figure 4:**
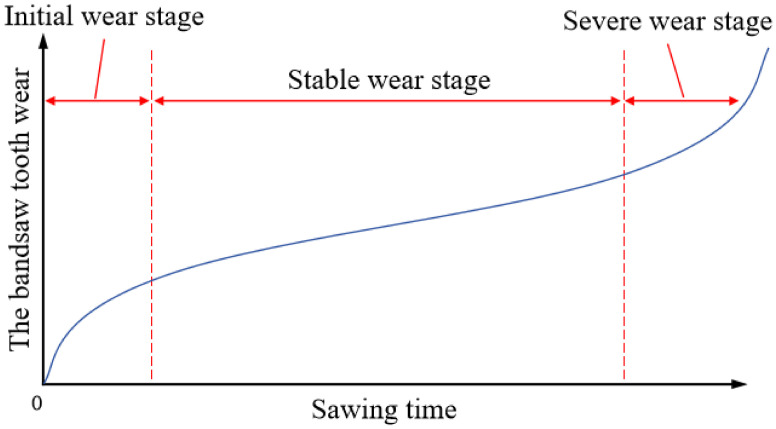
Typical wear evolution curve of a band saw blade based on the measurement of tooth tip wear width.

During the band sawing process, the saw teeth continuously rub and compress against the workpiece, generating heat. The combined effects of friction, compression, and high temperature lead to gradual wear. According to the classical tool wear curve, the flank wear VB increases with the increase in cutting length. The wear process of the band saw teeth can be divided into three stages: the initial wear stage, the stable wear stage, and the severe wear stage. The slopes of wear curves in the initial wear stage and the rapid wear stage are larger than the steady wear stage, which shows that the wear speed is faster. The wear rate for each of these stages is illustrated in Figure 4 below [23,24,25]. Following a brief period of rapid wear during the band sawing initial stage, the band saw tooth transitions into the stable wear stage, where the wear rate slows down. However, once it progresses to the severe wear stage, the wear rate of the band saw tooth accelerates, and tooth breakage typically occurs during this phase.

In the initial wear stage of the band saw blade, the tooth tips of both sides were observed to wear predominantly, while the back of the blade surface was worn minimally. This initial wear was primarily attributed to high-temperature oxidation of the high-speed steel layer on the surface. Wear near the tooth tip was caused by frictional contact between the processed surface and the band saw tooth’s front blade surface, resulting in the formation of metal chip build-up or tumor. The accumulation of chip tumors on the front blade surface had an impact on cutting conditions of the band saw tooth, including force and temperature. During this phase, the thrust and cutting force components acting on the band saw teeth increase with increasing wear. Once these force components reach an equilibrium, the band saw blade wear transitions into the stable stage, characterized by a slower growth rate of thrust and cutting forces [26]. During this period, the accumulation and decomposition of the built-up edge on the rake face continue; however, their influence on the cutting force gradually diminishes and even provides a certain degree of protection to the cutting edge. Furthermore, as tooth tip wear progresses, the cutting edge radius increases. When the feed rate remains constant and the radius of the cutting edge exceeds the thickness of the metal layer to be cut, the band saw tooth primarily removes metal material through a plowing action.

**Figure 5:**
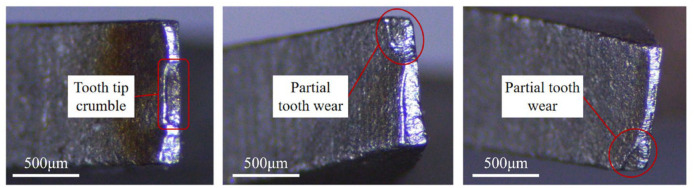
Severe wear patterns of the center tooth, left-set tooth, and right-set tooth of a band saw blade.

Figure 5 presents microscopic images of the band saw blade teeth at multiple positions along the blade, namely, the set tooth, the left-set tooth, and the right-set tooth. During the severe wear stage, which marks the end of the band saw blade’s service life, obvious material loss occurs at the tooth tip, accompanied by an increase in the cutting edge radius. This stage is characterized by uneven wear on the tooth side and notable asymmetric wear on the left and right deviated tooth, factors that contribute to a decrease in sawing stability. Consequently, the band saw blade becomes susceptible to deflection, which results in slanted cuts [27].

**Figure 6:**
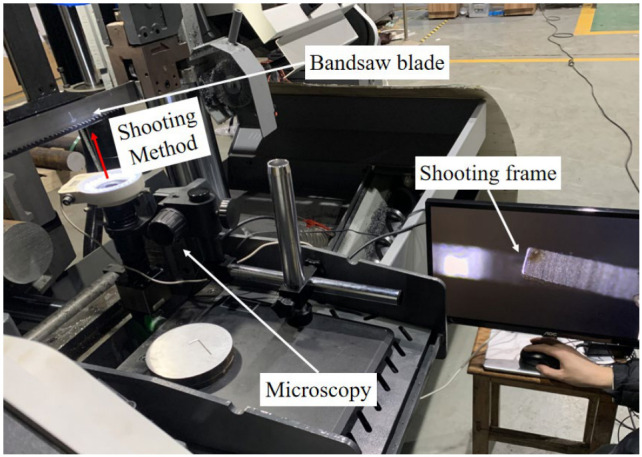
Method of photographing band saw tooth wear.

In order to establish a correlation between band saw tooth wear and current, it is essential to quantify the wear state of the tooth. Based on studies of band saw tooth wear, it is evident that increased wear severity leads to a larger radius of the cutting edge, which is the primary factor behind the augmented load on the band saw tooth. Consequently, this study integrates the expertise prevalent in sawing processes and the band saw blade wear assessment methodology employed by Bichamp Cutting Technology (Hunan) Co., Ltd. (Changsha, China) to devise a comprehensive evaluation framework for the band saw tooth wear condition. This framework utilizes the width of wear at the tooth tip as a quantitative metric and outlines the corresponding measurement technique. The measurement approach, illustrated in Figure 6, involves positioning the microscope beneath the band saw blade when it is elevated to its peak position on the sawmill. The microscope bracket’s angle is adjusted to ensure a perpendicular shooting angle relative to the band saw blade. The lens height is then fine-tuned until the rear blade surface near the tooth tip is in focus, enabling a clear shot and subsequent scale marking.

**Figure 7:**
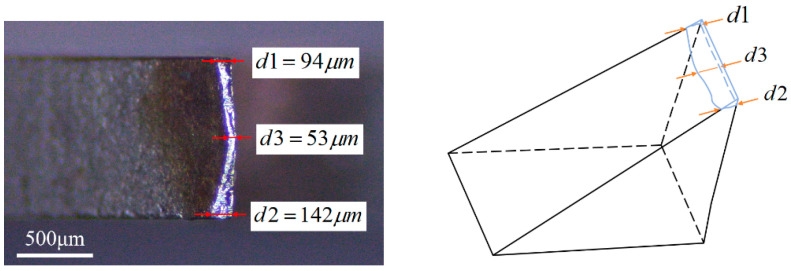
Measurement method for the tooth wear width VB.

Following photography, the lateral wear widths, designated as *d*_1_ and *d*_2_, along with the intermediate wear width, labeled as *d*_3_, of the band tooth were measured using the provided scale. Subsequently, the average of these three measurements was computed using Equation (6). This average was termed the average wear width *VB* of the serrated tooth tip. By averaging the results of three measurements, we can effectively minimize the impact of human error during the measurement process, resulting in values that more closely reflect reality. For instance, in Figure 7, *VB* is 96 μm. In this study, the band saw blade is currently in the stable wear phase.

(6) $$VB=\frac{d1+d2+d3}{3}$$

**Figure 8:**
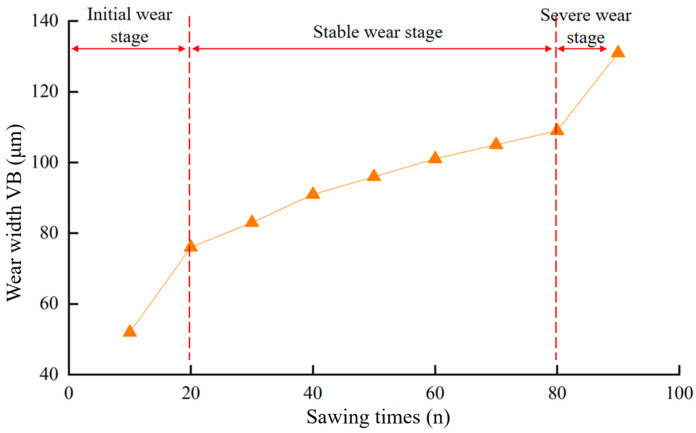
Statistics of wear width VB and division of wear stages in the full-life experiment of the saw blade.

The average wear width at the tip of the straight tooth is used as the indicator for evaluating the wear state of the band saw teeth. The chosen straight tooth wear serves as a proxy for the overall wear of the band saw blade. In this experiment, a 34 × 1.1−3/4Pt−10B high-speed steel bimetal band saw blade manufactured by Bichamp Cutting Technology (Hunan) Co., Ltd., Changsha, China. was used to cut 20# steel bars. Tooth wear data was collected and, based on the growth rate of wear amount depicted in Figure 4 for each stage of the high-speed steel bimetallic band saw blade, the wear of the experimental blade was categorized into three distinct stages. The orange triangles in the figure represent the tooth wear width values *VB* measured every 10 cutting cycles of the band saw blade, and the orange lines are the connections between two adjacent data points. as illustrated in Figure 8 below. This study uses the amount of sawtooth wear (*VB*) at points where the growth rate of the curve in the figure changes significantly as the boundary for distinguishing between different wear stages. The corresponding value ranges for each wear stage are detailed in Table 2.

## 3. Experiment Method

**Figure 9:**
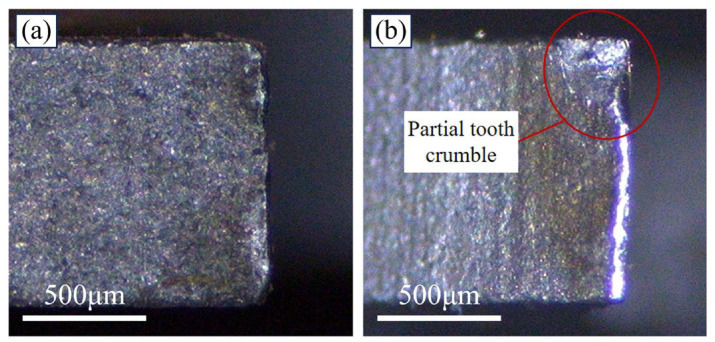
Comparison of tooth tip morphology after one cut with a run-in saw and an unrun-in saw. (**a**) First sawing after running-in; (**b**) First sawing without running-in.

During the actual band sawing process, selecting the appropriate band saw blade model is typically required, taking into account the size of the workpiece, material properties, and cross-section shape. Based on these considerations, machining efficiency and quality requirements should be comprehensively evaluated within the recommended range of cutting parameters in the machining manual. Once band sawing commences, the band saw blade linear speed remains constant, and operators often adjust the feed speed based on factors such as processed surface quality and noise. Currently, the tooth tips of HSS bimetallic band saw blades available in the market undergo sandblasting, although some may still retain minimal burrs, which can impact the blade’s cutting performance. Furthermore, the sharpness of a new band saw blade’s tooth tip can result in premature wear, as depicted in Figure 9b, when used at high cutting speeds initially, thereby significantly reducing the blade’s lifespan and compromising the quality of the machined surface. Consequently, it is imperative to sharpen the band saw blade prior to commencing official sawing operations on the workpiece. Numerous studies have demonstrated that adequate sharpening enhances the durability of the band saw tooth tips, improves cutting performance, and extends the blade’s lifespan [28,29,30,31].

Based on the enterprise’s production experience, the typical approach for band saw blade grinding involves setting the initial knife line speed and feed speed at 50% of the official sawing parameters. Subsequently, each knife increment is adjusted to match the official sawing parameters, with a grinding duration of at least 30 min. In this experiment, to expedite the wear process of the blades, we opted for higher cutting parameters. The specific experimental parameters employed are detailed in Table 3 below.

Once the parameters have been established, the experiment should be conducted by the following steps:

After establishing the experimental parameters, the procedure was conducted in a continuous and systematic manner. The band saw blade was first installed on the horizontal metal band saw machine by loosening and subsequently tensioning the saw wheel, after which the blade was raised to its highest position. The microscope system was then calibrated by adjusting the lens angle to ensure perpendicularity to the back surface of the saw tooth, followed by securing the bracket and fine-tuning the lens height to obtain a clear image of the tooth profile. Meanwhile, a reference straight tooth was marked to facilitate subsequent observations. The power analyzer was then connected to the three-phase current of the driving wheel motor via the electrical box, and the blade was allowed to run idle to verify the stability of the current signal and record the no-load current.

Subsequently, the workpiece was positioned to achieve a cutting width of approximately 5 mm, and cutting operations were carried out sequentially using parameters 1 through 5 to ensure sufficient sharpening of the blade. Thereafter, the band sawing process was performed under cutting parameter 6, during which the motor current of the driving wheel was recorded for each cut. After every ten cuts, the operation was interrupted, the blade was raised, and the saw wheel was rotated to align the marked tooth within the microscope’s field of view. The workpiece was then removed, and images of the tooth wear were captured. This sequence was repeated periodically until failure of the band saw blade occurred.

## 4. Results and Analysis

### 4.1. Raw Current Signal Analysis

Due to the periodic nature of AC electrical signals, data captured at a single point in time is insufficient to precisely represent the instantaneous current value. Consequently, in this study, low-pass filtering and regression analysis were employed to preprocess the raw current data. The function derived from the regression analysis is designated as the effective current value function for the sawing process, and the current extracted using this function is considered the effective current.

**Figure 10:**
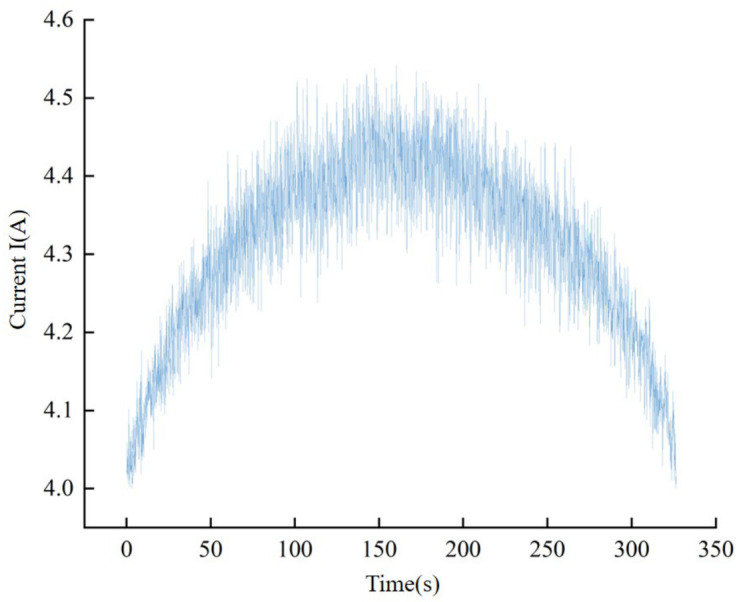
Raw current signal of the sawing process.

As illustrated in Figure 10, the collected current signals exhibit a distinct periodicity trait. This periodic variation is a consequence of the alternating current (AC) characteristics inherent to the driving wheel motor, where the root mean square (RMS) value of its three-phase current undergoes regular fluctuations over time. During the band sawing process, the current signal experiences brief yet pronounced fluctuations upon encountering hard spots within the workpiece. These fluctuations manifest as a sudden surge in current value. However, given the relatively scarce and uneven distribution of these hard points within the workpiece, such occasional and minor current anomalies are not indicative of the broader current analysis and should therefore be excluded from the data set. In essence, individual data points collected lack clear practical significance. To obtain a more precise representation of the current’s overall trend, it becomes imperative to functionalize the current data, thereby extracting the underlying patterns inherent to the current signal.

**Figure 11:**
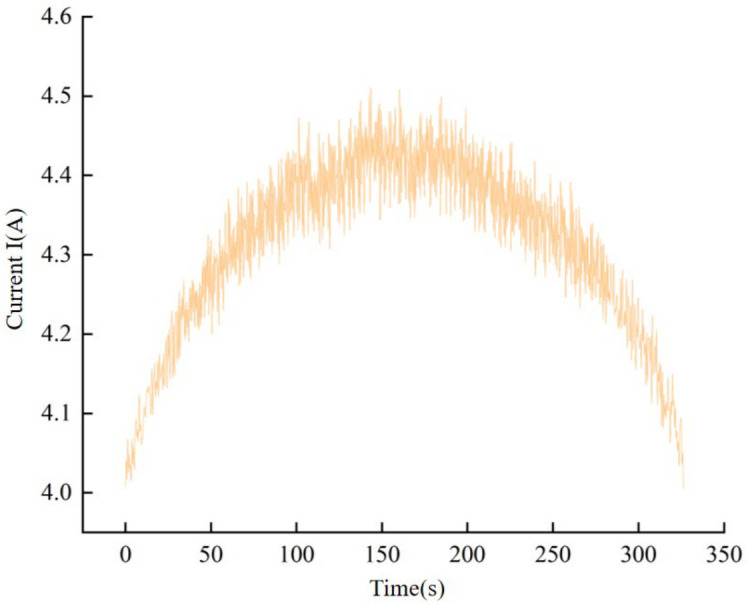
Removal effect of high-frequency noise after low-pass filtering of the raw current signal.

The filtering process is capable of efficiently extracting valuable signals from those contaminated with interference. During band sawing, the saw blade may encounter internal hard spots in the workpiece, resulting in instantaneous transients in the current signal. To extract the envelope representing the steady-state variations of the cutting load, a moving average low-pass filter was employed in this study. Considering the relatively low cutting speed of the band saw and a sampling frequency of 10 Hz, the filter window was set to five data points (corresponding to a time constant of 0.5 s). This parameter selection is based on the Nyquist sampling theorem and is intended to suppress random noise and transient current fluctuations while preserving the low-frequency features of the cutting load associated with blade wear. In this study, the collected current data underwent low-pass filtering prior to conducting regression analysis, as illustrated in Figure 11.

To extract the effective value from the periodically varying current signal after eliminating the interference current, it is necessary to establish a regression function that is tailored to the characteristics of the band sawing current. In the preceding section, a model was established to represent the relationship between current and cutting force, as expressed in Equation (5). Based on the mathematical relationship between sawing force, feed speed, and sawing cross-section width, Equation (5) can be transformed into a function of form I = f(t). This function enables regression analysis to determine the effective current during band sawing at a constant speed but with varying feed rates, as conducted in this experiment.

**Figure 12:**
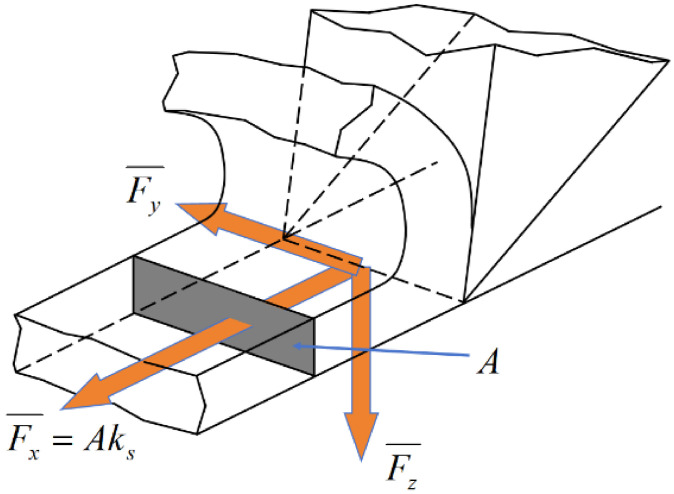
Classical source of metal cutting force.

The classical metal cutting theory proposed by Martellotti [32] posits that the normal cutting force exerted by the tool on the chip cross-section is equivalent to the multiplication of the undeformed chip area A and the sawing force specific pressure Ks (also known as the specific cutting pressure). This is depicted in Figure 12, the arrows in the figure indicate the directions of the corresponding forces. The formula for calculating the specific cutting pressure is presented below:

(7) $$k_{s}=K_{s}A^{\alpha }$$

where *K_s_* is the scale factor, *α* is the exponential coefficient, and the value is obtained by experiment. The undeformed chip area is calculated as [33]:

(8) $$A=b\frac{pV_{f}}{V_{c}}28$$

where b is the band saw blade width and *p* is the tooth pitch.

When the sawing line speed remains constant, according to the classical metal cutting theory and by combining Equations (7) and (8), it becomes evident that the cutting force exerted by a single tooth solely depends on the feed speed. The mathematical expression for this relationship is given as:

(9) $$\bar{F_{x}}=K_{s}V_{f}^{\beta }$$

The number of teeth simultaneously involved in the cutting process depends on the width of the workpiece cross-section and the tooth pitch. Consequently, a scale factor is incorporated to reflect the impact of cross-sectional width of the sawing zone on the cutting force. By integrating Equations (5) and (9), the present model is derived as follows:

(10) $$I=I_{M}+kdV_{f}^{\beta }$$

where *I_M_* is the no-load current; *d* is the saw section width; and the coefficients *k* and *β* need to be calculated from the experimentally collected data.

Since the raw current signals that are gathered constitute time-flow data, Equation (10) must be converted into a time-dependent function. Additionally, Equation (11) defines the chord length for a circle:

(11) $$d=2\sqrt{r^{2}-m^{2}}$$

**Figure 13:**
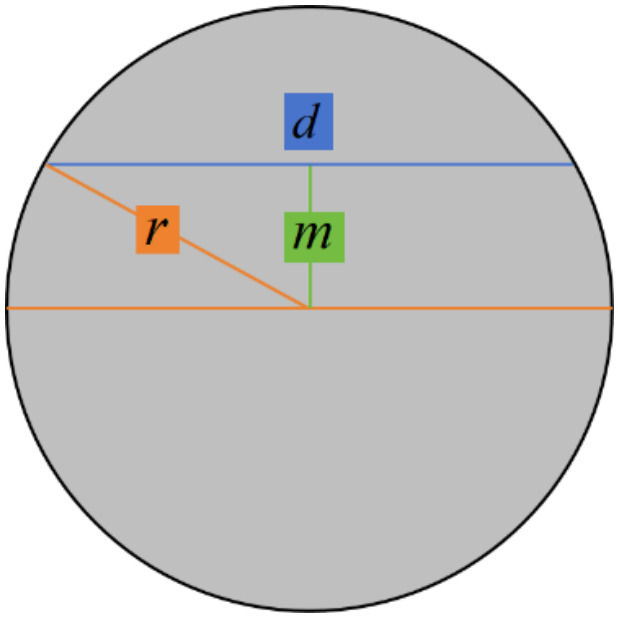
The cross-section of the workpiece.

Each parameter in the above Equation (11) is shown in Figure 13 below, where *d* is the section width, *r* denotes the radius of the circular cross-section, and m represents the distance from the tooth tip to the circle center.

In this experiment, the line speed and the feed speed of the band saw blade are held constant. Furthermore, the cross-section of the workpiece to be sawed assumes a circular shape, which necessitates the adoption of mm/s as the unit for measuring feed speed. By combining Equations (10) and (11), it is possible to ascertain the current as a function of time, given a particular feed speed.

(12) $$I=I_{M}+2kV^{\beta }\sqrt{DVt-{\left(Vt\right)}^{2}}$$

where *D* is the diameter of the workpiece circular cross-section.

The regression analysis of the experimentally acquired current data was conducted using Equation (12), which was derived from the preceding rationale. It was postulated that the obtained regression function represents the current behavior during sawing at a specific feed rate. Furthermore, the maximum current value, denoted as *I_max_*, was determined from this function and corresponds to the effective current when sawing reaches the maximum cross-section of the workpiece. This finding serves as the foundation for the current analysis conducted in this study.

### 4.2. Correlation Between the Amount of Wear and Current at Different Wear Stages

**Figure 14:**
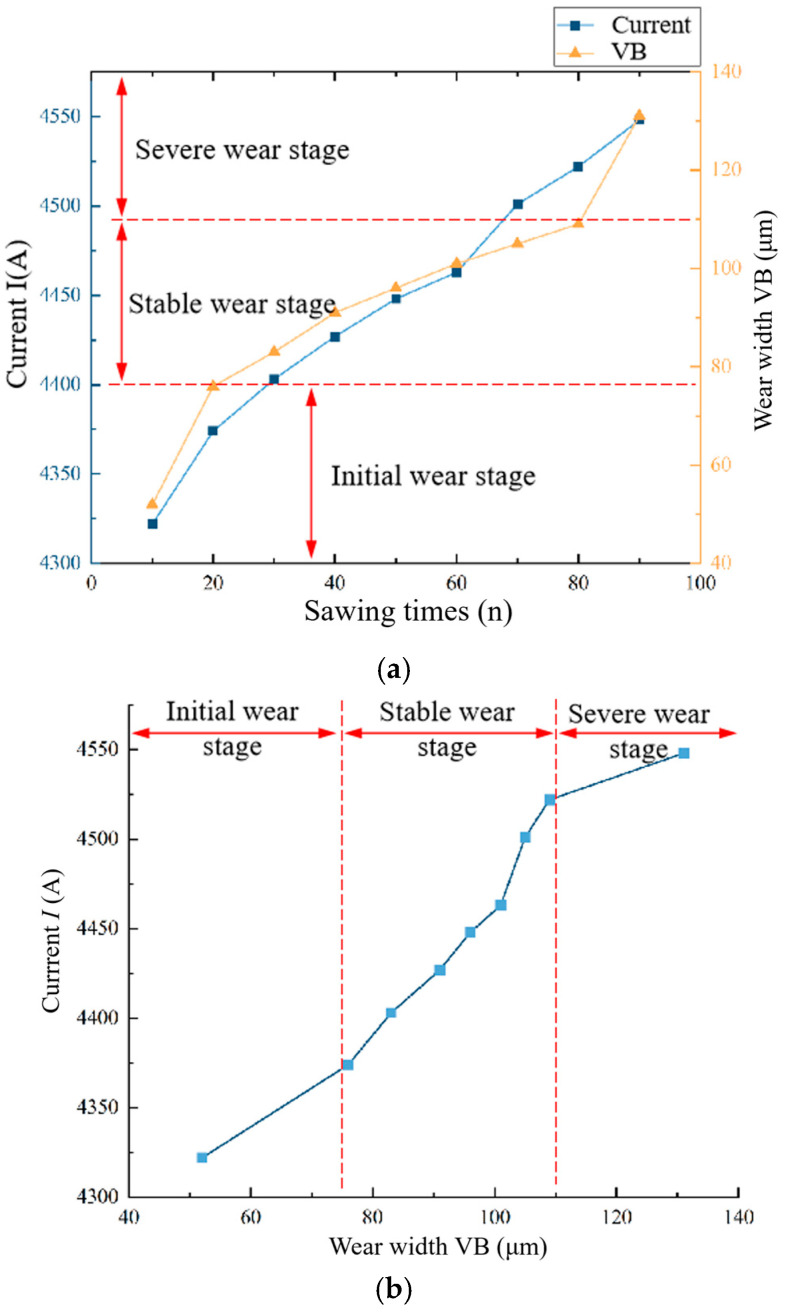
Relationship between the saw tooth wear and driving motor current. (**a**) Variation trends of tooth wear width and peak current during sawing. (**b**) Variation of maximum current with different flank wear widths VB.

The band saw blade’s wear state can be categorized into three distinct stages based on the extent of tooth wear. These are initial wear, steady wear, and severe wear. During the initial wear stage, the tooth exhibits excellent cutting capabilities, although the tooth tip experiences accelerated wear. As the wear transitions into the stable phase, any increase in cutting forces starts to plateau. Nevertheless, as the rate of material depletion from the tooth tip progressively rises and wear conditions worsen, the cutting efficiency of the band saw tooth undergoes a notable decline. Throughout this progression, the impact of band saw blade chatter and internal workpiece hard spots on cutting forces becomes increasingly significant. Considering that the cutting conditions of the band saw teeth differ with the stage of wear, the correlation linking tooth wear on the band saw to the drive wheel motor current also exhibits corresponding differences. The relevant data is presented in Figure 14.

As illustrated in Figure 14, a strong correlation exists between the driving wheel motor current and the wear degree of the band saw tooth. Specifically, as the band saw tooth wear increases, the motor current also rises commensurately. During the initial wear stage and the stable wear stage of the band saw blade, the magnitude of the drive wheel motor current exhibits a high correlation with the degree of the band saw tooth wear. However, this correlation diminishes in the severe wear stage. This reduction in correlation can be attributed to the significant variations in the wear state among individual band saw teeth following the stable wear stage. Consequently, relying on the wear amount of a single straight tooth to represent the overall wear state of the entire band saw blade becomes less accurate. Furthermore, as the band saw blade wear becomes more severe, the amplitude of vibration increases, thereby influencing the current characteristics during the band sawing process to some degree.

The degree of association between two elements can be quantified by the correlation coefficient, a widely utilized statistical indicator. This coefficient not only characterizes the closeness and linearity of the relationship between the variables but also signifies their mutual substitutability. Its mathematical expression is given as follows [34]:

(13) $$\rho_{XY}=\frac{\sum_{i=1}^{n}\left(x_{i}-\bar{x})(y_{i}-\bar{y}\right)}{\sqrt{\sum_{i=1}^{n}{\left(x_{i}-\bar{x}\right)}^{2}}\sqrt{\sum_{i=1}^{n}{\left(y_{i}-\bar{y}\right)}^{2}}}$$

where *x_i_* and *y_i_* are the *ith* sample values of the random variables *X* and *Y*, and $\bar{x}$ and $\bar{y}$ are the mean values of the random variables *X* and *Y*.

In statistical analysis, the strength of the linear correlation, as determined by the absolute value of the correlation coefficient, can be categorized into five levels: significant, high, moderate, low, and no correlation. The intensity of this linear correlation serves as a direct indicator of the mutual substitutability between two variables. To investigate the substitutability between the driving wheel motor current and saw tooth wear, this study computes the correlation coefficient between the two, thereby quantifying their degree of substitutability. The findings are presented in Table 4. An analysis of this table allows for evaluating the suitability of employing the driving wheel motor current as a proxy for band saw tooth wear, thus assessing its feasibility as an indicator.

The aforementioned analysis leads to the conclusion that the magnitude of the current of the drive wheel motor plays a significant role in assessing the extent of band saw tooth wear during the sawing process. However, it is crucial to observe that *I_max_* provides a more precise reference for wear measurement when the band saw tooth wear is minimal. Nevertheless, sawing stability diminishes as the wear amount progresses, resulting in augmented fluctuations in the current signal. Consequently, during such instances, the effective current approximated by the effective current function diverges significantly from the actual current, thereby reducing the accuracy of evaluating band saw tooth wear solely based on the value of *I_max_*.

**Figure 15:**
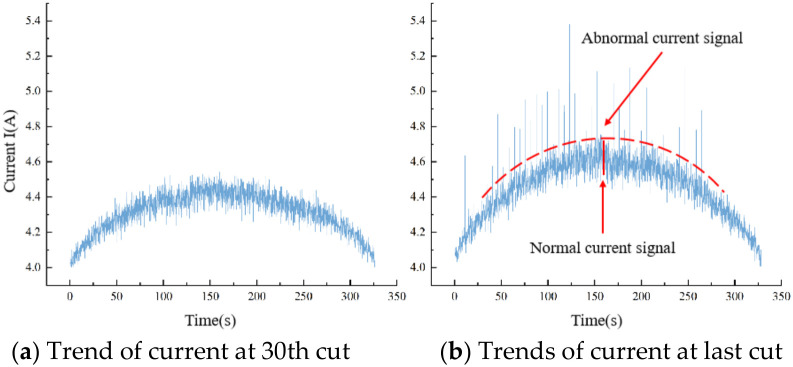
Comparison of current variation trends between the 30th cut and the last cut in sawing.

As shown in Figure 15, the stability of band sawing experiences a notable decline as the band saw blade approaches its failure state. This instability is reflected in the substantial growth of current fluctuations and the emergence of abnormal current signals. This deterioration arises from the dulling of a portion of the band saw blade’s teeth during extended use. When these dulled teeth engage in the cutting process, they trigger a sudden surge in cutting force, ultimately leading to abrupt variations in current.

**Figure 16:**
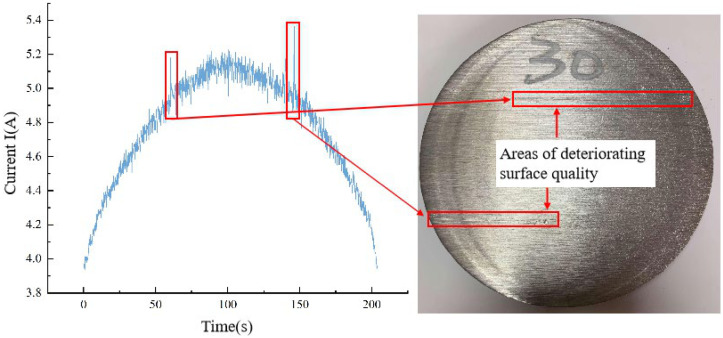
Relationship between surface deterioration area and current abrupt change.

The current signal serves not only to indicate the wear status of the band saw tooth but also to reflect defects on the machined surface. Referring to Figure 16, it is evident that whenever there is a deteriorated region on the machined surface of a workpiece, the current in that specific area undergoes significant and abrupt changes.

In conclusion, our findings indicate that during the initial stages of band saw tooth wear, current fluctuations remain within a relatively narrow range. Consequently, the error margin of the current value derived from fitting is minimal, allowing for a more precise reflection of the band saw tooth wear status. Sudden shifts in current during this phase are predominantly caused by internal hard spots within the workpiece. However, as the band saw blade approaches the end of its useful life and teeth begin to dull, the range of current fluctuations widens considerably. This widening is attributed to the combined effects of blade chatter and tooth dullness, resulting in an uptick of abnormal mutation currents. Consequently, the likelihood of tooth breakage rises during this period. To mitigate potential risks, we recommend adjusting the feed speed downward or halting the process entirely.

### 4.3. Parametric Model of Band Sawing with Unworn Band Saw Blade

The magnitude of the driving wheel motor current is influenced by several factors during the band sawing process. These include feed speed, line speed, the extent of band saw tooth wear, saw section width, workpiece characteristics, and the material properties of the band saw tooth. Previous research has established that, when cutting parameters remain constant, there is a strong correlation between the severity of band saw tooth wear and the increase in current observed during the sawing of a given workpiece. To design an evaluation system that uses the value of the current from the drive wheel motor to assess the degree of tooth wear, it is first necessary to construct a mathematical model that relates the feed rate of the unworn band saw blade, the cross-sectional width of the band saw cut, and the drive wheel motor current. This model can then be compared to the actual current measurements, serving as a basis for determining the amount of band saw tooth wear.

In order to conduct a band sawing experiment comprising 10 groups, as illustrated in Table 5, a consistent type of band saw blade and sharpening technique was employed across every experimental group. The cutting speed and feed rate selected for this experiment were based on the *Metal Band Saw Cutting Parameters Manual* provided by the band saw blade manufacturer (Bichamp Cutting Technology (Hunan) Co., Ltd.). For low-carbon steel materials, these parameters fall within the industrially recommended high-efficiency range. Although the higher parameter settings shorten the saw blade’s service life to facilitate experimental observation, the resulting cutting heat and mechanical stress characteristics still conform to the physical principles of industrial heavy-duty sawing. This ensures that the established current signal model covers wear monitoring requirements ranging from conventional machining to extreme production capacity conditions.

**Figure 17:**
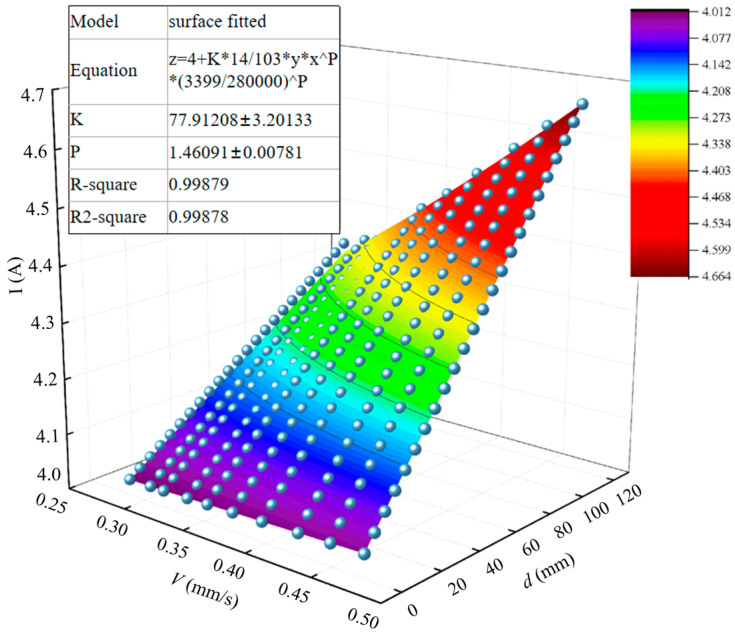
The sawing experiment *V*-*d*-*I* data surface fitted.

The three-dimensional fitting tool provided by Origin 2024 software is capable of generating various linear and nonlinear curves or surfaces. In our study, we conducted 10 experimental groups, where the sawing cross-section width ranged from 5 mm to 120 mm, with measurements taken at 5 mm intervals along with their corresponding current values. This implies that for each experimental group, 24 sets of *V*-*d*-*I* data were obtained, resulting in a total of 240 data sets. These data sets were then imported into Origin and subjected to nonlinear surface fitting using Equation (12) as the objective function. The resulting fitted surface is shown in Figure 17. The circles represent the original experimental data points.

The parameters *K* = 77.91208 and *β* = 1.46091 were determined through regression analysis of the fitted surface mentioned above. Incorporating these parameters and constant terms, the relational model of *V*-*d*-*I* can be formulated as follows:

(14) $$I=4+0.01303dV^{1.46091}$$

where *I* is the driving wheel motor current, *d* is the band saw section width, and *V* is the band saw blade feed speed.

The performance is influenced by several factors, including the characteristics of the band saw blade tooth, the nature of the workpiece material, the cutting temperature, and others. Consequently, the aforementioned solution formula applies only to the specific sawmill, band saw blade, and workpiece employed in this study. In practical applications, it is essential to evaluate the variations in these factors to guarantee the formula’s applicability and precision.

In statistics, *R*^2^ serves as a crucial measure of how accurately a fitted model represents a given data sample. Its fundamental idea revolves around determining the percentage of variance in the data set’s variables that can be attributed to the fitted curve. The *R*^2^ value ranges from 0 to 1, where an *R*^2^ near 0 signifies that the model barely explains the sample data, whereas an *R*^2^ approaching 1 signifies that the model nearly perfectly interprets the data. The regression model was formulated in a nonlinear form, (*I*= *I*_0_ + *k d*^β^ *V*^α^). The power-law form was chosen with reference to classical metal cutting force models, in which cutting force exhibits a power-law relationship with cutting thickness and width. The parameter (*k*) represents a coefficient related to material properties, while (β) and (α) denote the exponents reflecting the contributions of cutting width (*d*) and feed velocity (*V*) to the current, respectively. Regression fitting using the least squares method yielded an (R^2^) of 0.998, demonstrating that the model accurately represents the influence of workpiece cross-sectional geometry on the current signal.

### 4.4. Methods for Assessing the Wear Condition of the Band Saw Blade

**Figure 18:**
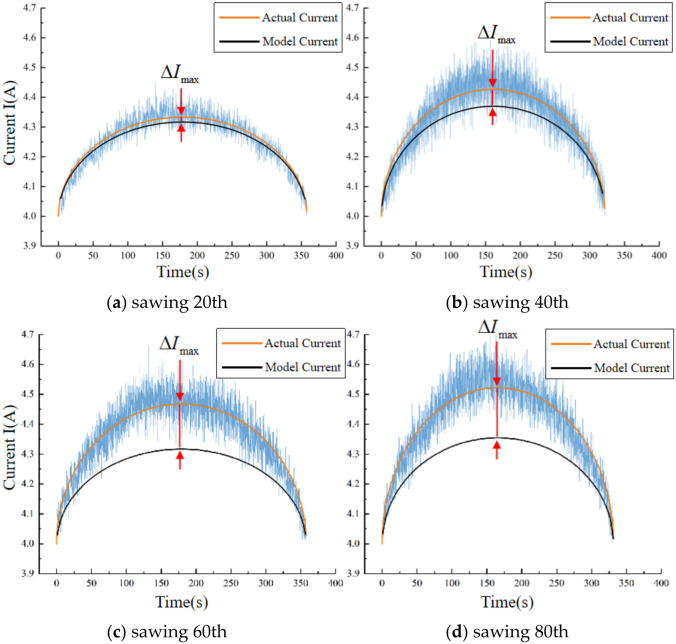
Comparative analysis of model current and actual current for the 20th, 40th, 60th, and 80th sawing cuts.

Prior experimental research into the wear condition of band saw blades has established a link between saw blade wear and the current drawn by the sawing machine’s drive wheel motor. This is assuming constant cutting parameters. To accurately evaluate the wear condition of the band saw tooth via variations in current, it is imperative to extract the information closely correlated with band saw tooth wear from the current signal. Given that the experimental current signal is a time-based data stream, the original Equation (14) has been transformed into a time-variable functional form. This allows for the construction of a comparative framework aligned with the current model. Assuming a constant feed rate during the machining of the blade and utilizing the formula for a circle’s chord length, the original Equation (14) can be reformulated into the following time-dependent functional expression:

(15) $$I=4+0.02606\sqrt{DVt-{\left(Vt\right)}^{2}}V^{1.46091}$$

where *D* is the diameter of the workpiece circular cross-section. A comparison between the sawing current model established in this study of the unworn blade condition and the actual current measured experimentally is shown in Figure 18. Herein, ∆*I_max_* is computed according to Equation (16).

(16) $$\Delta I_{\max}=n_{\max}KC_{t}VB$$

**Figure 19:**
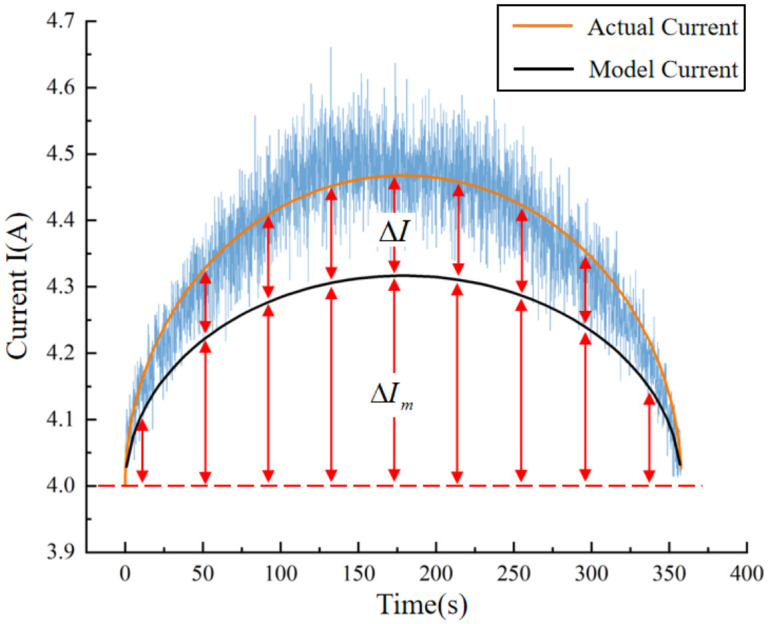
Definition of the sawing current ∆*I*_m_ and ∆*I*.

Upon analysis of the data presented in the aforementioned figure, a discernible pattern emerges: with increasing levels of band saw blade wear, the discrepancy between the actual current measured during sawing and the current model established in this study for the unworn state increases progressively. This observation provides a practical method for assessing the band saw teeth’s existing wear condition, which involves monitoring the deviation separating the actual effective current from the model current to determine the degree of wear. To gain further insight into the relationship between band saw tooth wear and the motor current of the driving wheel, we extracted relevant effective and model current data, which are graphically represented in Figure 19. Using this data, we calculated the difference between the currents, denoted as *I*, and the increment of the model current relative to the no-load current, designated as ∆*I*. As demonstrated in Equation (15), the formula for *I* and ∆*I* are derived in Equation (17).

(17) $$\left\{\begin{array}{l} \Delta I=V^{\beta }\sqrt{DVt-{\left(Vt\right)}^{2}}(2k-0.02606)\\ \Delta I_{m}=0.02606V^{\beta }\sqrt{DVt-{\left(Vt\right)}^{2}} \end{array}\right.$$

Previous studies have elucidated that ∆*I_m_* originates from the consistent sawing of circular cross-section workpieces using unworn band saw teeth at a fixed feed rate. Consequently, when the band saw tooth sustains wear, ∆*I* emerged. To objectively quantify the influence of band saw tooth wear on the current, we introduce the current growth ratio, denoted as *I_inc_*= ∆*I*/∆*I_m_*. This ratio indicates the proportion of the current increase in the worn state relative to the sawing current in the non-worn state. Consequently, the formula for *I_inc_* can be derived as follows:

(18) $$I_{inc}=\frac{2k-0.02606}{0.02606}$$

Equation (18) reveals that the magnitude of the parameter *I_inc_* is solely dependent on the fitting parameter *k*. This dependency arises from the fact that, when the saw blade and workpiece material remain constant, the unknown parameter *k* in the effective current fitting function is primarily influenced by the wear condition of the band saw tooth. Notably, this influence is feed speed-independent. In more precise terms, as the saw tooth wear increases, the value of *k* rises accordingly. Consequently, *I_inc_* serves as a reliable indicator for representing the wear of band saw teeth.

**Figure 20:**
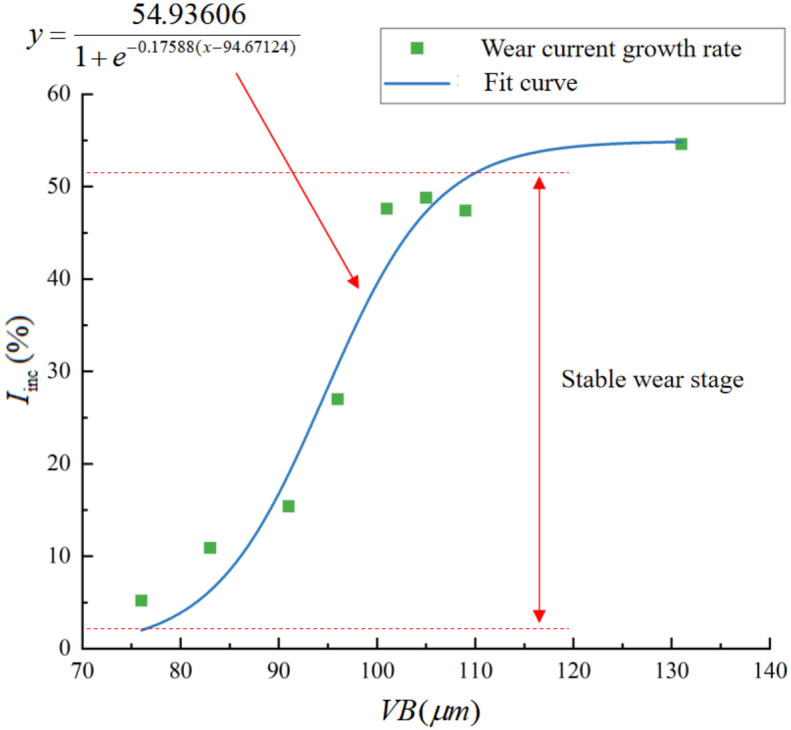
Relationship between wear and current growth rate.

In this study, we constructed an effective current fitting function based on the assumption that the degree of band saw tooth wear remains unchanged throughout the sawing process. With this premise, the unknown parameter *k* of our model stays consistent during each feed, implying that the ratio ∆*I*/∆*I_m_* is considered a constant value. To elucidate the correlation between the extent of band saw tooth wear and its corresponding parameter, we conducted a fitting analysis between *VB* and *I_inc_*. Furthermore, horizontal lines were plotted, passing through the points *VB* = 75 μm and *VB* = 110 μm on the fitting function, which intersect the y-axis. This delineates the distinct wear stages of the saw tooth. This visualization is presented in Figure 20.

Through the analysis of the above figure, it can be seen that once the saw blade enters the stable wear stage (*I_inc_* < 5), the wear rate of the band saw teeth gradually increases within this stage, as defined by *I_inc_*. The function curve trend for *VB* = f(*I_inc_*) is the same as that of *VB* = *f*(*t*). Table 6 displays the correlation analysis between *I_inc_* and *VB*.

*I_inc_* and *VB* exhibit a strong linear correlation in both the stable wear and severe wear stages, enabling the assessment of band saw blade wear based on the value of *I_inc_*. Using this experiment as a reference, the relationship between *I_inc_* and *VB* can be formulated using Equation (19):

(19) $$I_{inc}=\frac{54.93606}{1+e^{-0.17588(VB-94.67124)}}$$

By solving the inverse solution under known conditions, the corresponding band saw tooth wear can be determined. This model possesses a certain degree of predictive capability for band saw tooth wear. Table 7 presents the criteria for determining the band saw blade’s wear condition, according to the growth ratio of the driving wheel’s motor current, within the sawing conditions of the experiment. The wear evolution law of the band saw blade over its entire lifecycle and its corresponding electrical signal characteristics can be summarized into the following three stages:

Initial wear (VB ≤ 75 μm, *I*_inc_ ≤ 3%): The cutting edge rapidly loses its sharpness; due to the limited contact area, the increase in current is relatively small. The current exhibits a rapid rise at the commencement, corresponding to the sharp increase in cutting force caused by edge dulling prior to the formation of a stable wear zone.

Stable wear (75 < VB ≤ 110 μm, 3% < *I*_inc_ < 52%): A stable wear zone forms on the rake face, and the cutting force increases linearly with the width of the wear zone; the increase in cutting force is proportional to the contact area between the rake face and the machined surface. This linear relationship can be directly translated into a linear increase in current with wear amount VB, with a correlation coefficient of 0.92 between the two. In this stage, the current growth rate *I*_icn_ can serve as a reliable substitute indicator for wear amount VB.

Severe wear (VB ≥ 110 μm, *I*_inc_ ≥ 52%): The wear zone becomes excessively large, causing the cutting edge to fail and the cutting mechanism to shift from shearing to plowing. Uneven wear on left-hand and right-hand teeth exacerbates vibration and chatter, thereby introducing significant fluctuations in the current signal. The correlation coefficient between *I*_inc_ and wear volume VB is 0.95; however, due to the chipping of tooth tips and the random nature of vibrations, the relationship between the two becomes more dispersed. At this point, the current growth rate exceeds 52%, indicating that the saw blade is nearing the end of its service life.

## 5. Conclusions

This study is based on experiments investigating the wear of bimetallic band saw blades when cutting low-carbon steel. A mathematical model was derived relating the current of the band saw drive motor to the blade load, and a correlation was established between the current signal and the wear condition of the saw blade. The specific conclusions are as follows:

(1) It has been established that a direct correlation exists between the drive motor current and the resultant cutting force. For band saw blades that have not been used, this study proposes a current equation that incorporates both cutting width and feed rate. This establishes a mathematical model of the relationship between the current signal and the load on the band saw blade. The model demonstrates a high degree of precision in its description of the mathematical relationship between the drive motor current and the sawing parameters, as evidenced by a coefficient of determination (R^2^) of 0.99879.

(2) A quantitative relationship was established between the average tooth tip wear width, the current signal, and the wear stage. In this study, the service life of the band saw blade was divided into three stages: cuts 1 through 19 were classified as the initial wear stage, cuts 20 through 81 as the steady-state wear stage, and cuts 82 and beyond as the severe wear stage. During the initial and steady-state wear phases, a significant correlation was observed between the maximum current signal and the degree of tooth tip wear, with a correlation coefficient as high as 0.99. This indicates a strong linear relationship between the variables. Even during the later severe wear phase, the correlation coefficient remained consistently above 0.95.

(3) By comparing the actual measured current with the current predicted by the model under unworn conditions, a quantitative metric for evaluating the degree of wear was established. The wear evolution of the band saw blade can be divided into three stages. In the initial wear stage (VB ≤ 75 μm, Iinc ≤ 3%), the cutting edge rapidly becomes blunt; however, due to the limited contact area, the increase in current remains small. In the stable wear stage (75 < VB ≤ 110 μm, 3% < Iinc < 52%), a steady wear land is formed, and both cutting force and current increase approximately linearly with VB, making the current growth rate a reliable indicator of wear progression. In the severe wear stage (VB ≥ 110 μm, Iinc ≥ 52%), excessive wear leads to reduced cutting stability and a change in cutting mechanism, accompanied by increased vibration and significantly enhanced signal fluctuations. The rapid rise in current at this stage indicates that the saw blade is approaching the end of its service life.

## Figures and Tables

**Table 1:** Hardware equipment for band saw tooth wear experiment.

Equipment	Product Model	Performance Parameter
CNC horizontal metal band saw machine	Chenlong 330B	Motor power 4 kw
High-speed steel (HSS) bimetallic band saw blade	Bichamp 34 × 1.1−3/4Pt−10B	Length: 4115 mm, tooth width: 1.1 mm, tip material: high-speed steel, back material: spring steel
Three-phase power analyzer	YOKOGAWA WT330 (manufactured by Yokogawa Electric Corporation (China) Co., Ltd., Shanghai, China)	Accuracy: 0.1%
Metallographic microscope	OST-FB201 (manufactured by Suzhou OSTGUSNGXUE Optical Instrument Co., Ltd., Suzhou, China)	Zoom range: 25–200
Band sawing workpiece	Mild steel	Diameter: 120 mm

**Table 2:** Standard of the wear stage.

Wear Stage	Average Wear Width
Initial wear	≤75 μm
Stable wear	75 μm~110 μm
Severe wear	≥110 μm

**Table 3:** Parameters of saw tooth wear test.

Parameter Number	Feed Speed *V_f_* (mm/min)	Line Speed *V_c_* (m/min)
1	12	20
2	14	24
3	16	28
4	18	32
5	20	36
6	22	40

**Table 4:** Results of correlation coefficient.

Wear Stage	Correlation Coefficient	Correlation
Initial wear	0.99	significant linear correlation
Stable wear	0.99	significant linear correlation
Severe wear	0.95	high linear correlation

**Table 5:** Parameters of sawing experiment.

Group Number	Feed Speed *V_f_* (mm/min)	Line Speed *V_c_* (m/min)
1	0.2765	40
2	0.2950	40
3	0.3065	40
4	0.3270	40
5	0.3439	40
6	0.3648	40
7	0.3884	40
8	0.4167	40
9	0.4412	40
10	0.4661	40

**Table 6:** The results of correlation coefficient.

Wear Stage	Correlation Coefficient	Correlation
Stable wear	0.92	high linear correlation
Severe wear	0.95	high linear correlation

**Table 7:** Criteria for judging the wear stage of the band saw blade.

Wear Stage	Initial Wear	Stable Wear	Severe Wear
*I_inc_* (%)	≤3	3~52	≥52

## Data Availability

The original contributions presented in this study are included in the article. Further inquiries can be directed to the corresponding author.
